# Tri‐Parametric Assessment of α‐Galactosidase A Activity, lysoGb3 and X‐Inactivation Aids Genotype‐Phenotype Categorization of Fabry Disease Female Patients

**DOI:** 10.1002/jimd.70218

**Published:** 2026-06-22

**Authors:** Ladislav Kuchar, Lenka Dvorakova, Linda Berna, Martin Reboun, Petra Slavikova, Radovan Bakalar, Jana Ledvinova, Helena Poupetova, Petr Ruzicka, Asfaw Befekadu, Gabriela Dostalova, Stella Reichmannova, Ales Linhart, Jakub Sikora

**Affiliations:** ^1^ Research Unit for Rare Diseases, Department of Paediatrics and Inherited Metabolic Disorders First Faculty of Medicine, Charles University and General University Hospital Prague Czech Republic; ^2^ Diagnostic Laboratories of Inherited Metabolic Diseases, Department of Paediatrics and Inherited Metabolic Disorders First Faculty of Medicine, Charles University and General University Hospital Prague Czech Republic; ^3^ Second Department of Internal Cardiovascular Medicine First Faculty of Medicine, Charles University and General University Hospital Prague Czech Republic; ^4^ Institute of Pathology, First Faculty of Medicine, Charles University and General University Hospital Prague Czech Republic

**Keywords:** AGALopathy, alpha‐galactosidase A, Fabry disease, genotype‐phenotype correlation, lysoGb3, X‐chromosome inactivation

## Abstract

Fabry disease (FD, OMIM 301500) is an X‐linked lysosomal storage disorder caused by deficient activity of lysosomal alpha‐galactosidase A (AGAL, E.C. 3.2.1.22) due to pathogenic variants in the *GLA* gene (HGNC:4296, Xq22.1). Plasmatic deacylated globotriaosylceramide (lysoGb3) is elevated in FD patients as a reflection of lysosomal accumulation of Gb3. Specific (AGALopathic) *GLA* variants have been recently shown to accumulate within the secretory pathway and trigger endoplasmic reticulum stress and unfolded protein response rather than result in profound enzymatic deficiency. In part due to lack of integrative measures of clinical severity and biochemical/molecular parameters, specific impacts and consequences of X‐chromosomal inactivation (XCI) on clinical manifestation in FD female heterozygotes still remain to be fully understood. Our study aimed at evaluation of XCI (% of inactive wt GLA allele) in untreated female FD heterozygotes with classic FD (*n* = 17), late‐onset FD (*n* = 19) and individuals carrying *GLA* variants (p.(L394P) (*n* = 7), p.(A143T) (*n* = 4), and p.(D313Y) (*n* = 4)) with predominant AGALopathic effects. XCI was correlated with age of the patients, clinical phenotype, (residual) AGAL activity, and lysoGb3. AGAL activity corresponded to XCI independently of the type of the *GLA* mutation. The best separation of the clinical phenotypes (classic FD, late‐onset FD and AGALopathy) was achieved by correlating XCI to the ratio of AGAL activity to lysoGb3. This three parametric calculated marker was then confronted with the Mainz Severity Score Index (MSSI) to generate an Integrative Clinical‐Laboratory quotient (ICLq). ICLq discriminated the three female patient groups and demonstrated group‐dependent differences in its average age‐related increase.

## 1 Introduction

Fabry disease (FD, OMIM 301500) is a X‐linked lysosomal storage disorder caused by deficient activity of lysosomal alpha‐galactosidase A (AGAL, E.C. 3.2.1.22) due to pathogenic variants (mutations) in the *GLA* gene (HGNC:4296, *Xq22.1*) [1]. Major glycosphingolipid AGAL substrates include globotriaosylceramide (Gb3), digalactosylceramide (diGalCer) and lipids bearing the antigenic blood group B determinant [2]. Uncleaved metabolites accumulate in lysosomes and trigger abnormal metabolic and signaling cascades.

Classic FD is associated with cutaneous angiokeratomas, cardiomyopathy, premature cerebrovascular events and renal insufficiency in X‐hemizygous male patients. Additional symptoms include acroparesthesia, angiokeratomas, hypohidrosis and/or hyperhidrosis, gastrointestinal symptoms, and lymphedema [3]. A subset of patients presents with late‐onset FD often limited to single organ involvement (cardiac and/or renal). Females heterozygous for *GLA* mutations are symptomatic, however, their FD phenotype is variable and delayed in time [4, 5] in comparison to male patients.

Endoplasmic reticulum (ER) stress and unfolded protein response (UPR) have been suggested to result from several missense *GLA* variants [6, 7]. AGALopathy, as a pathogenic mechanism stemming from retention of the mutant AGAL in the early secretory pathway (ER and ER‐Golgi intermediates) and consequent ER stress and UPR, has been proposed by our group based on linkage and functional studies in several families with the p.(L394P) AGAL/*GLA* variant and chronic renal disease/failure lacking the sub‐cellular lysosomal storage features of FD. We have demonstrated similar (AGALopathic) effects in other *GLA* variants as well. The current concept of Fabry disease pathogenesis and phenotypic diversity, therefore, reflects the likely contribution of both enzymopathic and AGALopathic molecular mechanisms that are differentially triggered by individual *GLA* variants [8, 9, 10].

FD laboratory diagnostics build on in vitro measurements of residual AGAL activity and molecular‐genetic testing in the *GLA* gene. Deacylated Gb3 (lysoGb3) has been established as a useful FD plasma biomarker that reflects the primary intracellular storage of Gb3 [11, 12, 13, 14, 15, 16]. Similar to AGAL activity testing, lysoGb3 measurements have major diagnostic value and predictive capacity in male patients with classic FD [17, 18, 19]. We have recently introduced the ratio of AGAL activity in plasma to plasma lysoGb3 as a novel calculated marker that facilitates diagnostic identification of classic and late‐onset male FD patients, and individuals with *GLA* variants with predominant AGALopathic effects [9, 17]. Utility of these biochemical tests (including the calculated ratio) remains, however, unsettled in heterozygous female FD patients [14, 15, 17, 19].

Mainz severity score index (MSSI) was introduced in 2004 [20]. A modified version of the score that utilizes only dichotomous variables was developed for the Fabry Outcome Survey (FOS‐MSSI) [21]. Neither MSSI nor FOS‐MSSI reflect the genetic FD aspects (e.g., type of a *GLA* variant). In 2010, a study by Hughes et al. [22] outlined mathematical models of age‐ and sex‐dependent clinical FD progression to allow inter‐individual comparisons in observed and predicted FOS‐MSSI scores.

X‐chromosome inactivation (XCI) [23, 24] is likely the key factor constituting the phenotypic variability in FD female patients [25, 26, 27, 28]. XCI ratios are, however, difficult to interpret as a stand‐alone measure for many biological reasons [29]. Moreover, limited size [27] and pooling of treated and untreated patients [26, 30] in tested cohorts, or methodological pitfalls of XCI assessment and interpretation [31] altogether constitute further potential sources of bias. As a result, studies on links of XCI and age, FD disease severity scores or levels of individual biochemical markers have, so far, presented variable results with some authors suggesting [25, 26, 32] correlations and others disputing [33, 34, 35, 36] them.

To address the current ambiguity, we tested whether comprehensive multilevel analyses of age, (residual) AGAL activity, lysoGb3, XCI (assessed as percentage (%) of the inactive *wt GLA* allele), and MSSI (or MSSI‐derived) scores allow prediction of the clinical phenotype and/or disease severity in untreated FD female heterozygotes.

## 2 Material and Methods

### 2.1 Patients

The cohort consisted of 51 untreated Czech female heterozygotes with mutations in the *GLA* gene. Females were divided into three distinct groups according to mutations associated with the particular phenotype in males as follows: classic FD (*n* = 17), late‐onset FD (*n* = 19), and individuals with *GLA* variants (p.(L394P) (*n* = 7), p.(A143T) (*n* = 4), and p.(D313Y) (*n* = 4)) that have predominant AGALopathic effects (combination of high residual enzymatic AGAL activity, predominant non‐lysosomal localization, and triggered ER stress and UPR as shown in our recent study [9]). The latter group is described as “AGALopathy” or “AGALopathic group” in the entire manuscript.

Phenotypic and molecular data (*GLA* variants and XCI) and age are summarized in Table 1. Patient numbers correspond to data descriptors in the Figures and in the Supporting Information.

**TABLE 1:** Phenotype, genotype, XCI values, and age of untreated female FD heterozygotes.

No.	Phenotype	Variant/predicted effect on the protein	XCI assay (ratio): active mut % to active wt %	Skewing	Age	AGAL (WBC)^a^	lysoGb3^b^	AGAL/lysoGb3^c^
1	Classic	c.1034dup, p.(S345Ffs*30)	83/17	**Skewed**	61	20.6	16.8	1.2
2	Classic	c.881T>G, p.(L294*)	38/62	Random	38	32.5	11.2	2.9
3	Classic	c.194 + 2T>C, p.(S65Rfs*61)	50/50	Random	34	32.5	16.2	2.0
4	Classic	c.801 + 3A>G, p.(L268Vfs*4)	23/77	**Skewed**	38	42.9	6.4	6.7
5	Classic	c.801 + 3A>G, p.(L268Vfs*4)	50/50	Random	46	38.2	9.2	4.1
6	Classic	**c.511G>C, p.(G171R)**	43/57	Random	35	18.1	5.3	3.4
7	Classic	**c.146G>C, p.(R49P)**	35/65	Random	49	35.4	9.3	3.8
8	Classic	**c.277G>A, p.(D93N)**	40/60	Random	19	23.2	9.2	2.5
9	Classic	**c.787A>C, p.(N263H)**	80/20	**Skewed**	56	8.1	24.7	0.3
10	Classic	**c.463G>C, p.(D155H)**	39/61	Random	37	25.4	11.8	2.2
11	Classic	**c.1133G>T, p.(C378F)**	57/43	Random	63	28.8	12.5	2.3
12	Classic	**c.281G>A, p.(C94Y)**	33/67	Random	29	23.8	17.2	1.4
13	Classic	**c.973G>A, p.(G325S)**	54/46	Random	22	22.6	3.6	6.2
14	Classic	**c.239G>T, p.(G80V)**	75/25	**Skewed**	23	12.2	15.3	0.8
15	Classic	**c.239G>T, p.(G80V)**	81/19	**Skewed**	18	13.4	8.2	1.6
16	Classic	**c.1025G>A, p.(R342Q)**	54/46	Random	31	12.3	14.4	0.9
17	Classic	**c.1025G>A. p.(R342Q)**	34/66	Random	31	24.7	5.1	4.8
18	Late‐onset	**c.902G>A. p.(R301Q)**	79/21	**Skewed**	75	13.9	5.2	2.7
19	Late‐onset	**c.868A>G. p.(M290V)**	45/55	Random	23	25.1	1.8	13.6
20	Late‐onset	**c.454 T>C. p.(Y152H)**	58/42	Random	33	19.8	2.5	7.8
21	Late‐onset	c.801 + 48T>G. p.(L268Vfs*4)	10/90	**Skewed**	35	41.7	1.3	32.1
22	Late‐onset	c.801 + 48T>G. p.(L268Vfs*4)	38/62	Random	40	46.2	1.9	24.5
23	Late‐onset	c.801 + 48T>G. p.(L268Vfs*4)	57/43	Random	20	47.4	1.0	49.9
24	Late‐onset	c.801 + 48T>G. p.(L268Vfs*4)	67/33	Random	68	18.2	1.7	10.8
25	Late‐onset	c.801 + 48T>G. p.(L268Vfs*4)	79/21	**Skewed**	47	12.3	1.1	11.4
26	Late‐onset	c.801 + 48T>G. p.(L268Vfs*4)	79/21	**Skewed**	36	18.1	2.1	8.8
27	Late‐onset	c.801 + 48T>G. p.(L268Vfs*4)	78/22	**Skewed**	71	25.6	2.1	12.1
28	Late‐onset	**c.644A>G. p.(N215S)**	81/19	**Skewed**	64	11.4	4.3	2.6
29	Late‐onset	**c.644A>G. p.(N215S)**	71/29	Random	28	12.9	1.6	8.1
30	Late‐onset	**c.644A>G. p.(N215S)**	25/75	**Skewed**	58	35.4	1.3	27.1
31	Late‐onset	**c.644A>G. p.(N215S)**	65/35	Random	19	17.6	1.4	12.9
32	Late‐onset	**c.644A>G. p.(N215S)**	82/18	**Skewed**	42	12.5	4.2	3.0
33	Late‐onset	**c.644A>G. p.(N215S)**	64/36	Random	38	18.6	2.3	8.3
34	Late‐onset	**c.644A>G. p.(N215S)**	48/52	Random	46	24	1.6	14.7
35	Late‐onset	**c.644A>G. p.(N215S)**	79/21	**Skewed**	68	19.3	2.7	7.2
36	Late‐onset	**c.644A>G. p.(N215S)**	34/66	Random	30	N.A.	1.4	N.A.
37	AGALopathy	**c.427G>A. p.(A143T)**	22/78	**Skewed**	21	46.3	1.1	42.5
38	AGALopathy	**c.427G>A. p.(A143T)**	21/79	**Skewed**	35	62.4	0.9	72.6
39	AGALopathy	**c.427G>A. p.(A143T)**	60/40	Random	30	38.8	0.7	57.8
40	AGALopathy	**c.427G>A. p.(A143T)**	82/18	**Skewed**	39	25.5	1.0	25.5
41	AGALopathy	**c.1181T>G. p.(L394P)**	11/89	**Skewed**	46	54.2	0.8	66.9
42	AGALopathy	**c.1181T>G. p.(L394P)**	18/82	**Skewed**	56	67.9	0.9	77.3
43	AGALopathy	**c.1181T>C. p.(L394P)**	62/38	Random	28	20.5	1.7	12.1
44	AGALopathy	**c.1181T>C. p.(L394P)**	44/56	Random	56	50.3	0.8	63.4
45	AGALopathy	**c.1181T>C. p.(L394P)**	24/76	**Skewed**	51	44.5	1.5	28.9
46	AGALopathy	**c.1181T>C. p.(L394P)**	10/90	**Skewed**	51	68.5	1.6	41.9
47	AGALopathy	**c.1181T>C. p.(L394P)**	57/43	Random	39	25.6	0.8	31.3
48	AGALopathy	**c.937G>T. p.(D313Y)**	47/53	Random	29	51.4	0.8	65.1
49	AGALopathy	**c.937G>T. p.(D313Y)**	66/34	Random	50	45.3	0.5	96.4
50	AGALopathy	**c.937G>T. p.(D313Y)**	35/65	Random	49	49.5	0.7	76.2
51	AGALopathy	**c.937G>T. p.(D313Y)**	58/42	Random	60	47.5	1.1	44.8

*Note:* Females #4 and #5 carried the c.801 + 3A>G *GLA* variant resulting in insertion of 36 bases from intron 5 [25] and causing the classic FD phenotype. Females #21–27 carried the c.801 + 48T>G variant, which triggered insertion of 66 bases from intron 5 and caused late‐onset phenotype [37]. AGALopathy phenotype was assigned based on clinical presentation and the type of the GLA variant according to Zivna et al. [9].

^a^ AGAL activity in peripheral white blood cells (WBC)—nmol.mg^−1^.h^−1^, (24.8–103 nmol.mg^−1^.h^−1^, 59.7 ± 14.6, *n* = 477).

^b^ lysoGb3 in plasma—pmol/mL (0.2–1.73 pmol/mL, 0.8 ± 0.39, *n* = 30) [15].

^c^ AGAL/lysoGb3 represents ratio of AGAL activity in WBCs to plasma lysoGb3—nmol.mg^−1^.h^−1^/pmol.mL^−1^ (26.6–158 nmol.mg^−1^.h^−1^/pmol.mL^−1^, 78.6 ± 30.8 *n* = 21) [15].

### 2.2 Determination of X‐Chromosomal ( *GLA* ) Inactivation

Genomic DNA (gDNA) was extracted from the peripheral white blood cells (WBCs) (QIAamp DNA Blood Mini Kit DNA, Qiagen). Complementary DNA (cDNA) was reverse transcribed (High Capacity RNA to cDNA Kit, Applied Biosystems) from the total WBC RNA (BiOstic Blood Total RNA Isolation Kit, MO BIO Laboratories).

X‐chromosome inactivation (XCI) was determined using the following two different techniques—(i) gDNA was used for testing the methylation status at two polymorphic repeat regions located in *AR* (Xq12) and *RP2* (Xp11.3) genes [38, 39]; (ii) cDNA was used for the allele‐specific expression (ASE) assay of two heterozygous common SNPs—rs1141608 and rs12097 in *IDS* (Xq28) and *LAMP2* (Xq24) genes, respectively. The ASE assay was similarly used for the analyses of relative expression of the wild‐type and mutated *GLA* alleles. Amplicon sequencing (NEXTERA XT) on the Illumina MiSeq platform was applied.

Determination of the preferentially inactivated allele was based on segregation analyses of a male proband and/or other family members, on results of *GLA* transcript analysis and/or AGAL enzyme assay. A more detailed description of the method is available in Reboun et al. [31].

Presented XCI values reflect percentage (%) of inactivation of the *wt* allele of the *GLA* gene, values in the ranges ≤ 25/75 or ≥ 75/25 (*wt*/*mut*) were considered skewed (Table 1).

### 2.3 AGAL Activity Evaluation in WBCs and Plasma

AGAL activity in peripheral WBCs and plasma was measured by fluorometric method using 4‐methylumbelliferyl‐α‐D‐galactopyranoside as a substrate (final concentration 2.5 mM). N‐acetylgalactosamine (final concentration 0.1 M) was used as an inhibitor of α‐D‐galactosidase B in the WBCs assay [31, 40]. For measurements of AGAL activity in plasma, the method published by Merta et al. was used [41].

### 2.4 Plasma lysoGb3 Quantification

Plasma samples were processed and analyzed according to a method by Kuchar et al. [17]. The method is based on liquid–liquid extraction of lysoGb3 using organic solvents and water to create a two‐phase system. Internal standard for mass spectrometry lysoGb3 quantification was added to samples during the extraction process. Samples were analyzed using isocratic liquid chromatography on normal phase coupled to tandem mass spectrometry.

### 2.5 Calculation of lysoGb3 Exposure and the Tri‐Parametric Calculated Marker (TCM)

Linear regressions of lysoGb3 and age of females separated to classic, late‐onset, and AGALopathy groups were calculated. Intercept in each group represents average lysoGb3 concentration for the females in the particular group at birth. If the lysoGb3 value in the female was below the intercept for the group, then the life lysoGb3 exposure was calculated by multiplying lysoGb3 concentration by age of the patient. If the lysoGb3 value was above the intercept, then the following equation was used (for more details see Supporting Information).

$$\text{life}\;\text{exposure}=\left(\text{intercept}\times \mathrm{age}\right)+\frac{\left(\text{lysoGb}3-\text{intercept}\right)\times \mathrm{age}}{2}$$

To simplify the assessment, we also calculated the tri‐parametric calculated marker (TCM) as the ratio of AGAL activity in WBCs to plasma lysoGb3, divided (corrected) by XCI.

$$\mathrm{TCM}=\frac{\left(\frac{\text{AGAL activity in WBCs}}{\text{lysoGb}3\;\text{in plasma}}\right)}{\text{fractional}\;\mathrm{wt}\;\mathrm{GLA}\;\text{allele expression in WBCs}}$$

As an example, “fractional wt GLA allele expression in WBCs” in a female with 80% of inactivation of the *wt* GLA allele was 0.2.

### 2.6 MSSI, FOS‐MSSI, and Age‐Adjusted FOS‐MSSI (∆FOS‐MSSI)

All females included in the study underwent clinical evaluation during a short hospital stay. Mainz severity score index (MSSI) values were calculated based on detailed personal and family history questionnaire and clinical assessment [20].

FOS‐MSSI scores for each patient were re‐calculated based on the MSSI values [21]. These FOS‐MSSI scores were considered as “observed” for further calculations. Predicted FOS‐MSSI score for each patient was based on the following formula: [22]

$$\text{predicted}\;\text{FOS‐MSSI}={\left[0.96+\left(0.05\times \mathrm{age}\right)\right]}^{2}$$

Age‐adjusted FOS‐MSSI (∆FOS‐MSSI) was calculated as follows:

$$\text{ΔFOS‐MSSI}=\left(\text{observed}\;\text{FOS‐MSSI}\right)-\left(\text{predicted}\;\text{FOS}\text{‐MSSI}\right)$$

### 2.7 Genetic + MSSI Score (gMSSI+) and Integrated Clinical‐Laboratory Quotient (ICLq)

To allow the calculation of the Integrative Clinical‐Laboratory quotient (ICLq) values, all total MSSI scores were increased by the value of one (gMSSI+) to reflect the presence of a *GLA* variant.

ICLq was then calculated as:

$$\text{ICLq}=\frac{\text{gMSSI}+}{\mathrm{TCM}}$$

### 2.8 Statistical Analyses

The datasets in the Figures were evaluated either in whole or separated into classic FD, late‐onset FD, and AGALopathy groups. Linear regressions/regression coefficients (*R* ^2^) were used to summarize the correlations. If not stated otherwise, an unpaired two‐tailed Student *t*‐test was used to assess the differences in phenotypic groups based on the calculated *p* value. *p* ≤ 0.05 was set as the minimal value to confirm differences between the compared groups. All calculations were performed using MS Excel.

OriginPro 2018 was used for calculation of ROC curves as previously described [17]. Outliers in the data were identified using OriginPro 2018 as values below or above the range defined as 1.5× of the interquartile range (IQR).

Data are listed as average ± standard deviations.

## 3 Results

### 3.1 XCI ( *GLA* Inactivation) Status in the Patient Cohort

Clinical phenotypes in the patient cohort were categorized according to Germain et al. [42] and Zivna et al. [9] and correlated to particular mutations and phenotypes observed in affected X‐hemizygous male relatives. The whole cohort of 51 untreated females (Table 1) consisted of 17 patients with mutations associated with classic FD phenotype and 19 with late‐onset FD. In the late‐onset FD group, 3 patients carried private mutations, 7 patients were heterozygotes for the c.801 + 48T>G, and 9 patients for the p.(N215S) *GLA* variant (Table 1). The cohort further included female heterozygotes carrying *GLA* variants with predominant AGALopathic effects [9] (AGALopathy group)—7 carrying p.(L394P), 4 carrying p.(A143T), and 4 carrying p.(D313Y) *GLA* variants. There were 22 different *GLA* variants in the entire cohort.

Skewed XCI (≤ 25/75 or ≥ 75/25 range) was detected in 21 out of the total number of 51 tested females (41.2%). Individuals with skewed XCI constituted 29.4% and 47.4% of patients in the classic and late‐onset FD groups, respectively. The fraction of skewed XCI was 46.7% among females with p.(L394P) and p.(A143T) *GLA* variants. XCI was not skewed in any of the females carrying the p.(D313Y) variant.

### 3.2 Correlations of Age With XCI and lysoGb3

The cohort consisted of females aged 18–75 years. The ages between the three phenotypic groups—classic FD (37 ± 14 years), late‐onset FD (44 ± 18 years), and AGALopathy (43 ± 12 years)—were not statistically different (Figure S1A).

Neither overall (*R* ^2^ = 0.03) nor phenotypic group specific (classic *R* ^2^ = 0.0308, late‐onset *R* ^2^ = 0.1255, AGALopathy *R* ^2^ = 0.025) correlation was detected between the XCI (% of inactive *wt GLA* allele) values and age (Figure S1B).

**FIGURE 1:**
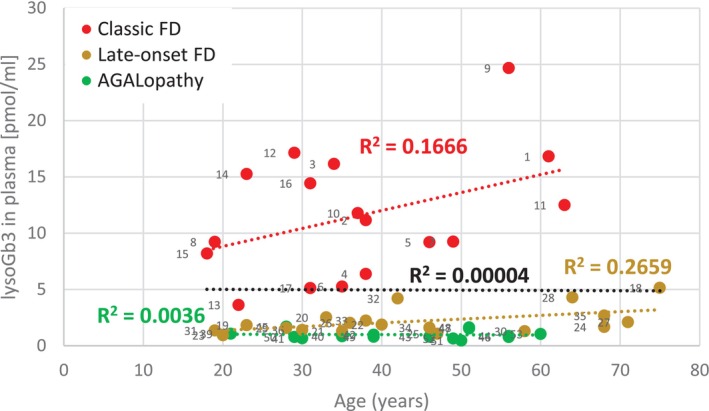
Correlation of lysoGb3 and age. Plasmatic lysoGb3 levels did not change with age (black dotted line) when calculated for all females regardless of their phenotype. Nonetheless, an increase was detected in the classic FD group. While not as steep as in the classic group, a relatively tighter correlation of the two parameters than in classic FD patients was seen in late‐onset FD patients. Patients carrying AGALopathic *GLA* variants had constant lysoGb3 levels independent of age. Regression line equations and their intercepts used for lysoGb3 exposure calculation were as follows: Classic: *y* = 0.1595x + 5.6395; late‐onset: *y* = 0.0334x + 0.7; AGALopathy: *y* = −0.0018x + 1.0625. As an important comparison: The intercept values 0.96 and 0.82 in a group of 7 patients with the c.801 + 48T>G and 9 patients with the p.(N215S) variant, respectively, were comparable to the intercept 0.7 value calculated in the entire group of late‐onset patients. The mean of ages in both groups of late‐onset FD patients carrying the two specific variants was ~50 years (Figure S14A,B). LysoGb3 values remained constant with age of patients carrying the AGALopathic variants (Figure S14C–E).

An increase of plasmatic lysoGb3 with age was identified in the classic FD group followed by the late‐onset FD group. In contrast, no increase in lysoGb3 levels with age was detected among individuals with AGALopathic *GLA* variants (Figure 1).

### 3.3 Correlations of XCI, AGAL Enzymatic Activity and lysoGb3

**FIGURE 2:**
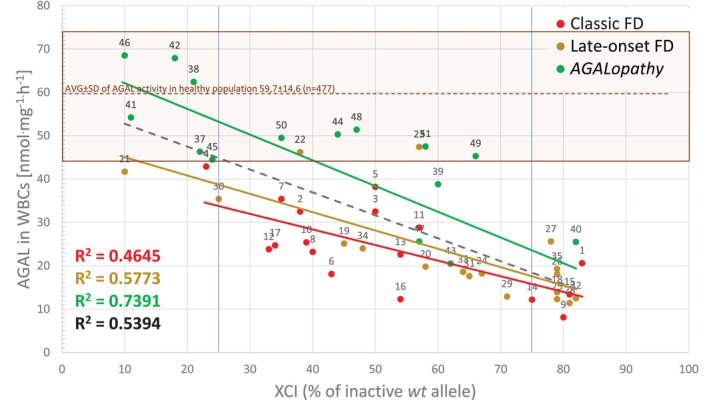
Correlation of AGAL activity in WBCs and XCI. AGAL activity decreases with inactivation of the *wt GLA* allele. The decrease is independent of the clinical phenotype (black dashed line represents regression for the entire cohort). Similar correlation patterns with higher *R* ^2^ values (Figure S4) were detected when individually analyzing values in females carrying late‐onset p.(N215S) and c.801 + 48T>G and AGALopathic *GLA* variants p.(L394P), p.(A143T) and p.(D313Y). Vertical blue lines highlight 25% and 75% of the inactive *wt GLA* allele. XCI was also analyzed in urinary cells [43] of 46 females from our cohort. Correlation of the urinary XCI with AGAL activity in WBCs was substantially weaker with correlation coefficient *R* ^2^ = 0.1688 (individual data points are not shown).

When measured in plasma, AGAL activity slightly decreased with inactivation of the *wt GLA* allele. However, the widely scattered values are only loosely or not correlated (*R* ^2^ = 0.1317, Figure S2). While AGAL activity in WBCs decreased more rapidly in all three groups in a much tighter (*R* ^2^ = 0.5394) XCl‐dependent manner (Figure 2), separation of the three phenotypes was still not possible. Importantly AGAL activity in WBCs of patients with AGALopathic *GLA* variants was always higher than 20 nmol.mg^−1^.h^−1^, which is about 30% of normal activity in healthy controls.

**FIGURE 3:**
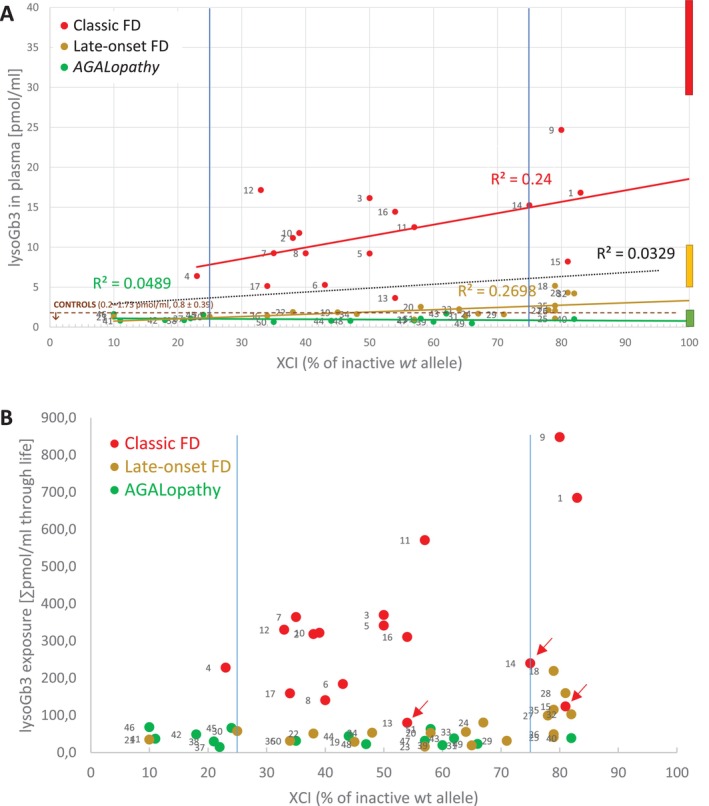
Correlation of lysoGb3 and lysoGb3 exposure with XCI. (A) Increase of lysoGb3 with inactivation of the *wt GLA* allele was evident in FD females with classic and late‐onset phenotypes. There is an overlap of lysoGb3 values in females with late‐onset FD and AGALopathic variants who had XCI values ≤ 25% of the inactive *wt* allele. Comparable correlation patterns (Figure S4) were detected when separately analyzing values in females with late‐onset p.(N215S) and c.801 + 48T>G and AGALopathic p.(L394P), p.(A143T), and p.(D313Y) *GLA* variants. Reference ranges for plasma lysoGb3 in male FD patients are shown in red, orange, and green boxes on the right. Control levels (brown dashed line) of lysoGb3 correspond to our previously published data [17]. (B) Correlation of lysoGb3 exposure with XCI. Values reflect the lifetime exposure to lysoGb3 by combining parameters shown in Figure 1. LysoGb3 exposure increases with the fraction of the inactive *wt GLA* allele in patients with the classic FD and to a lesser extent also in patients with late‐onset FD. Contribution of age to the calculated values can be demonstrated in patients #13 and #15 with the classic FD, who have not been exposed to the disease for so long. Patients #14 and #15 are another example of the impact of age (#14 is 5 years older than #15). Although they have the same genotype (p.(G80V)), their lysoGb3 values are substantially different. Patients #13, #14, and #15 are marked by arrows. Classic FD *R* ^2^ = 0.2021; Late‐onset FD *R* ^2^ = 0.2457, AGALopathy *R* ^2^ = 0.0708. LysoGb3 exposure remains constant irrespective of XCI among patients carrying AGALopathic *GLA* variants. Vertical blue lines highlight 25% and 75% of the inactive *wt GLA* allele.

Partial separation of female patients with the three different clinical phenotypes was allowed by correlating lysoGb3 to XCI (Figure 3A). The steepest increase of lysoGb3 was associated with the inactivation of the *wt GLA* allele in the classic FD group. Despite not being as distinct, the lysoGb3 increase with XCI was also evident in females with late‐onset FD. In contrast, females with AGALopathic variants did not show any changes in lysoGb3 relative to the inactivation of the *wt GLA* allele, representing a specific group distinguishable from the two other FD categories.

LysoGb3 values of some FD females with late‐onset FD overlapped with values detected in heterozygotes with AGALopathic variants with lower fractions of inactive *wt GLA* allele. Despite that, they differed in correlation of the two parameters (*R* ^2^ = 0.2698 and *R* ^2^ = 0.00489 in late‐onset and AGALopathic groups, respectively).

Clear separation from the other categories was achieved only in the classic FD group. LysoGb3 levels in the classic and late‐onset categories did not reach the reference values of plasmatic lysoGb3 in male FD patients (Figure 3A).

LysoGb3 exposure [16] is a derived parameter calculated using lysoGb3 concentration and age (Figure 3B). Females carrying *GLA* variants associated with classic FD had the highest increase of lysoGb3 exposure with increased inactivation of the wt *GLA* allele. LysoGb3 exposure values calculated in females with late‐onset FD overlapped with values in females with AGALopathy variants in the regions of inactivation favoring expression of the wild type (*wt*) *GLA* allele and random XCI. Growth of lysoGb3 exposure was seen in late‐onset patients with inactivation skewed towards increased inactivation of the *wt* GLA allele (Figure S3). Life exposure to lysoGb3 showed constant values in AGALopathy group independent of XCI.

**FIGURE 4:**
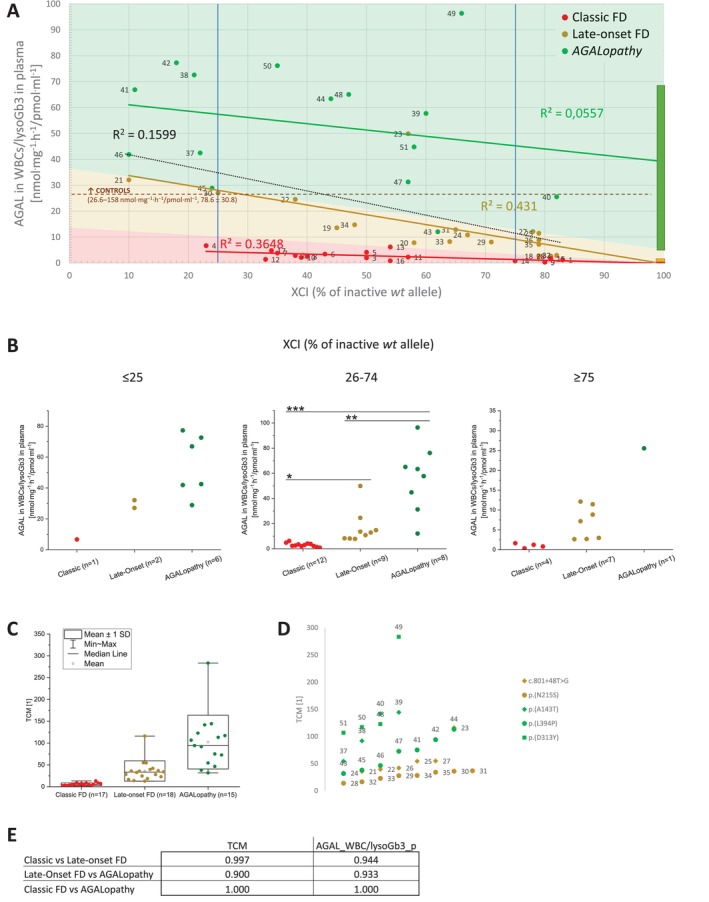
Tri‐parametric correlation—AGAL activity in WBCs/lysoGb3 in plasma ratio compared to XCI. (A) Separation of the three phenotypes was observed over the whole range of XCI deciles. Reference ranges for AGAL activity in WBCs/lysoGb3 in plasma ratio in male FD patients are shown in the boxes on the right. Control levels (brown dashed line) of the ratio correspond to previously published data [17]. Vertical blue lines highlight 25% and 75% of inactive *wt GLA* allele. Values in patients #21–#23 were identified as outliers. (B) Individual ratio values from A were plotted for ≤ 25%, 26%–74%, ≥ 75% ranges of the inactivated *wt GLA* allele. Note the differing scaling of the ratio values shown on the y axis in the three plots. Pair‐wise comparisons were not performed for ≤ 25% and ≥ 75% ranges because of the insufficient number of patients in these groups. *—unpaired two‐tailed Student *t*‐test *p* < 0.05; **—unpaired two‐tailed Student *t*‐test *p* < 0.01; ***—unpaired two‐tailed Student *t*‐test *p* < 0.001. As a note, the presented findings were supported by lowest total categorization error calculated for samples using linear discriminant analysis (data not shown). For comparison, high Gb3 degradative capacity, high residual AGAL activity, low plasma lysoGb3 and their relatively high ratio in males carrying p.(L394P) *GLA* variant in comparison to values in classic FD patients have been previously reported in Kuchar et al. [17] and Zivna et al. [9]. (C) Comparison of TCM (AGAL in WBCs/lysoGb3 in plasma/fractional *wt GLA* allele expression in WBCs) values for the three phenotypic groups. (D) Detailed comparison of distribution of TCM values in patients with late‐onset and AGALopathic GLA variants. Individual variants are color/symbol coded. There is just one overlapping TCM value (female #13, refer to panel A) between the classic and late‐onset groups. Note that there was no overlap between TCM values identified in patients with late‐onset FD *GLA* variants and individuals with p.(A143T) and p.(D313Y) *GLA* variants. (E) Specificity and selectivity of the AGAL activity in WBCs/lysoGb3 in plasma ratio values and TCM values to differentiate the phenotypic groups was comparable when assessed by calculating areas under ROC curves. Interestingly, separation of late‐onset and AGALopathy groups was improved (area under curve 0.982) by excluding all 7 patients carrying the c.801 + 48T>G intronic *GLA* variant due to an outlier status of AGAL activity in WBCs/lysoGb3 in plasma ratio values in patients #21–#23.

We further tested correlations between XCI values and the ratio of AGAL activity in WBCs to plasma lysoGb3. We previously demonstrated that this calculated value better reflects the metabolic dysfunction in FD [17] compared to isolated measurements of AGAL activity and/or lysoGb3. We used AGAL activity in WBCs for the calculation because it correlated substantially better with XCI than AGAL activity measured in plasma (Figures 2 and S2). The interaction of the three parameters is illustrated in Figure 4A,B representing the continuum of XCI and groups categorized by XCI range, respectively. The individual groups showed different linear regression characteristics, significantly improving the relation of the female phenotypic groups to their respective males' reference values. Interestingly, the linear regression for females with classic and late‐onset FD intersects the *x*‐axis at approximately the same point (97% and 99%) (Figure 4A, red and yellow lines). Similar patterns with higher *R* ^2^ coefficients for AGAL activity, lysoGb3 in plasma and their ratio were seen in patients with p.(N215S), p.(L394P), p.(A143T) and p.(D313Y) *GLA* variants (Figure S4).

Finally, we normalized the ratio of AGAL activity in WBCs to plasma lysoGb3 by the fractional *wt* *GLA* allele expression in WBCs to calculate the TCM values and showed its effectiveness to differentiate the three phenotypic groups (Figure 4C–E).

### 3.4 Comparison of MSSI‐Derived Scores With Age, XCI and Biochemical Characteristics in Individual Patients

MSSI, (observed) FOS‐MSSI and ΔFOS‐MSSI values for all females are summarized in Table S1. MSSI and FOS‐MSSI data tightly correlated (Figure S5A). Majority of the total MSSI (and FOS‐MSSI) values were < 20, which corresponds to the mild range of the scale. Comparisons showed a wide distribution of values with the highest average values in the classic, followed by late‐onset and AGALopathic groups in both scores (Figure S5B,C). There was no statistically significant difference between any two groups. Similarly, ΔFOS‐MSSI scores did not statistically differ between any two of the three groups; however, average values were above zero in patients with the classic and below zero in patients with late‐onset and AGALopathic *GLA* variants (Figure S5D).

Overall increase of MSSI (and FOS‐MSSI) with age was seen in the entire cohort; however, the correlation was minimal. Out of the three phenotypic groups, the tightest correlation was seen in late‐onset patients. Correlations of MSSI values with age within categorized XCI groups (≤ 25%, 26%–74%, ≥ 75% of the inactive *wt GLA* allele) showed an increase in all female groups. Similar to Hughes et al. [22], the distribution of ΔFOS‐MSSI scores became wider with age in all three groups (Figure S6).

MSSI values increased with XCI (% of the inactive *wt GLA* allele) in all three groups. The main contributors to this increase were, however, scores in the AGALopathic group (Figure S7A). Importantly, the only significantly different phenotypic group was females with XCI of the *wt* allele ≤ 25% who had MSSI values ~3 times lower than females with random XCI or inactivation of the *wt* allele ≥ 75% (Figure S7B). Similarly to MSSI, ΔFOS‐MSSI scores showed a tendency to increase with inactivation of the wild‐type *GLA* allele predominantly in the AGALopathic group (Figure S7C). Trends seen between MSSI and XCI and ΔFOS‐MSSI and XCI in the entire cohort were maintained among individuals with identical *GLA* genotypes. The tightest increases were identified in patients with AGALopathic p.(L394P) and p.(A143T) *GLA* variants (Figure S8).

MSSI values decreased with increasing AGAL activity in WBCs only in the AGALopathic group. These two parameters did not correlate in the two other phenotypic groups (Figure S9). While lysoGb3 levels in plasma helped to discriminate the three phenotypic groups (Figure 3), MSSI values did not systematically change with this biochemical parameter (Figure S10). As a likely contribution of the values of AGAL activity in WBCs, an overall decreasing trend of MSSI with increasing AGAL in WBCs/lysoGb3 in plasma ratio was detected in the cohort (Figure S11). Important, these associations were slightly stronger when tested in patients sharing the same *GLA* genotypes both for MSSI (Figure S12) and ΔFOS‐MSSI (Figure S13).

**FIGURE 5:**
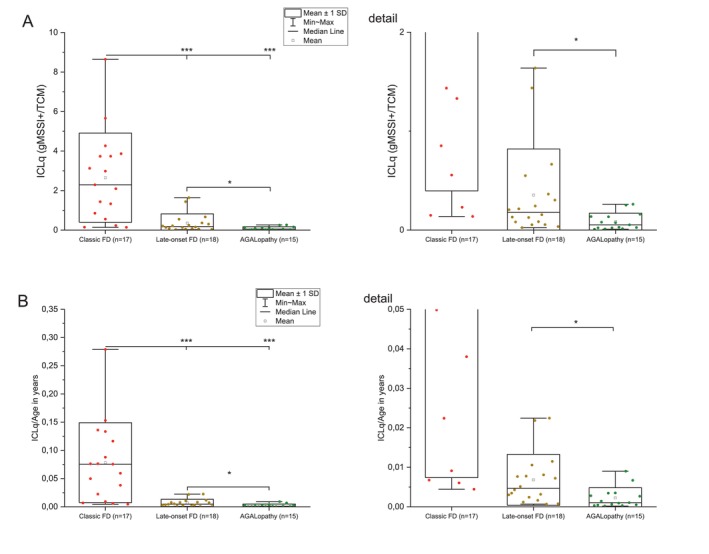
Integrative Clinical‐Laboratory quotient. MSSI scores in the female cohort were modified (gMSSI+) by increasing the original values by 1 in all patients based on the presence of the detected *GLA* variant. Integrated Clinical‐Laboratory Quotient (ICLq) was calculated as gMSSI+/TCM (A). Annual increase of ICLq for a given age (ICLq/age) (B). Compare the differences between the three groups to Figure S15 in which the ICLq was calculated using differentially weighted values for *GLA* variants based on their phenotypic impacts. *—unpaired two tailed Student *t*‐test *p* < 0.05, ***—unpaired two tailed Student *t*‐test *p* < 0.001.

gMSSI+ scores were established to avoid zero numerator values in the calculation of the Integrated Clinical‐Laboratory quotient (*ICLq = gMSSI+/TCM*). Figure 5A, shows statistically significant differences of ICLq between all three phenotypic groups. We further calculated an average annual increase of ICLq (Figure 5B). Statistical differences between the three phenotypic groups were again significant, with the highest values observed in the patients with classic *GLA* variants, followed by those with late‐onset and AGALopathic variants.

## 4 Discussion

Fabry disease is traditionally regarded as a lysosomal enzyme deficiency resulting in substrate storage that is considered the key trigger of the cellular, tissue and organ pathologies [1, 11]. Long‐term research efforts on understanding ER‐related storage‐independent pathogenic mechanisms in FD [6, 7, 44] have recently coalesced and a novel pathogenic mechanism—AGALopathy [8, 9, 10]—was suggested.

Majority of FD females become symptomatic. The onset of symptoms is delayed in many of them [21] with some never developing disease‐associated symptoms even at an older age. Parameters predicting manifestation and progress of the disease have not yet been fully identified. XCI mosaic tissue expression of *GLA* mutation(s) is likely the key biological mechanism for this variability/differences. Despite contradictive literary interpretations of XCI links to disease severity and progress, the accepted concept claims Gaussian distribution of XCI ratios among symptomatic FD females [25, 26, 30, 33, 35].

Available biomarkers (Gb3 and lysoGb3) have limited utility to categorize FD female patients to phenotypic groups [14, 15, 19]. Therefore, we and others [45] have proposed to use the AGAL activity to plasma lysoGb3 ratio [17]. This calculated measure reflects the functionality of the mutated enzyme in relation to the abnormal metabolite and distinguishes male patients with different FD phenotypes (including carriers of AGALopathic variants). However, only partial discrimination is possible in female patients [17].

Aiming to separate the clinical phenotypes, our study tested correlations of the three key parameters (XCI, AGAL activity, and lysoGb3) in 51 females heterozygous for *GLA* variants. The cohort consisted of patients with classic FD, late‐onset FD, and individuals with p.(L394P), p.(A143T), and p.(D313Y) *GLA* variants that have been associated with a predominant AGALopathic molecular mechanism [9]. Critically, to maintain consistency of the clinical and biochemical data, none of the participants had been treated with any therapies targeting or ameliorating the primary molecular FD defect. To allow analyses of continuous (and not categorized) variables from identical biological material, peripheral blood samples were collected and XCI ratios were assessed and calculated to reflect the % activation/inactivation of the two alternative *GLA* alleles. The fraction of the active mutant *GLA* allele (% of the inactive *wt GLA* allele) was used as a parameter representing XCI. We also checked for and corrected for interpretation pitfalls such as crossing‐over [31]. AGAL activity was measured both in WBC homogenates and plasma. LysoGb3 was tested in plasma. Correlations were calculated in the entire cohort, the three phenotypic groups, and also among patients carrying identical *GLA* genotypes (p.(N215S), c.801 + 48T>G, p.(L394P), p.(A143T), and p.(D313Y)) when their number was ≥ 4.

XCI ratios in blood may change with age in healthy women [46, 47]. Information about this phenomenon is, however, limited in FD. Wagenhauser et al. [30] reported stable patterns for at least 2 years. Our earlier study [31] showed XCI changes in 2 out of 8 patients over the time span of 3–15 years. While not statistically different (Figure S1A), classic FD females in our cohort had the lowest mean age, whereas the AGALopathic group had the highest average age. Similar to data published by others, XCI ratios were near‐Gaussian with skewed XCI detected in ~40% (29.4% of patients with the classic form, 47.4% with the late‐onset form, 46.4% with p.(L394P) or p.(A143T) *GLA* variants) of females in the cohort. The highest ratio 83/17 favoring expression of the mutant *GLA* allele and 10/90 for the active *wt GLA* allele determined the skewing extremes in both directions. Overall, our data do not imply any significant age‐dependent XCI change favoring the mutant *GLA* alleles (Figure S1B). However, we identified a group of 4 females aged 64+ years with late‐onset FD and XCI skewed in that direction.

Increase in lysoGb3 levels with age was reported insignificant in FD males but significant in females [16, 48]. This change is thought to be a result of disease progression, possibly due to a gradual increase in primary Gb3 accumulation. In our cohort, the age‐related increase trend was detected in classic and late‐onset FD females but could not be identified in patients with AGALopathic variants. Plasma lysoGb3 stayed constantly low in the latter group (Figures 1 and S14).

Residual AGAL activity is one of the key diagnostic parameters in male FD patients, but the interpretation of the values is problematic in females. We have shown earlier that *wt GLA* allele expression in females heterozygous for *GLA* mutations, which do not result in non‐sense mediated mRNA decay, correlates with inactivation of the *wt* allele and AGAL activity measured in WBCs corresponded to expression [31] of the *wt GLA* allele. Moreover, it was suggested that the type of the *GLA* variant or *GLA* mRNA stability do not determine AGAL activity values in FD females, but differ based on their categorized XCI status (≤ 25%, 26%–74%, and ≥ 75% of inactive *wt GLA* allele) [26, 31]. Our current study shows that contrary to plasma (Figure S2) AGAL activity in WBCs nearly linearly correlates with XCI (*R* ^2^ = 0.5394, Figure 2) in all three phenotypic groups as well as in patients with identical *GLA* variants. Importantly, AGAL activity in WBCs in AGALopathic patients was never below 20 nmol.mg^−1^.h^−1^ representing 30% of the activity of healthy controls.

Next, we addressed correlation of plasma lysoGb3 levels to XCI as previously suggested by *GLA* gene direct methylation study [32]. In our cohort, we found that the percentage of inactive *wt GLA* allele determined the levels of plasma lysoGb3 and clinical phenotype of the disease. The highest values of lysoGb3 were identified in the group of classic FD females with the highest proportion of the inactive *wt GLA* allele. An increase in lysoGb3 with increased percentage of the inactive *wt GLA* allele was also found in the group of late‐onset FD females. However, the increase was lower than in the classic FD group. In lower inactivation scores of the *wt GLA* allele, there was some overlap of lysoGb3 values between patients with late‐onset FD and AGALopathic variants. In the latter patient group, there was no increase in lysoGb3 even in females with higher % of inactivated *wt GLA* allele. Comparison with males showed that lysoGb3 levels in FD females do not converge to high lysoGb3 values in males as the plot vectors for FD females were below the range of values for males (Figure 3A).

Previously reported lysoGb3 exposure calculated as lysoGb3 multiplied by age was found to correlate with disease severity [16]. We modified the lysoGb3 exposure calculation to better reflect lysoGb3 elevation with age. Correlation of lysoGb3 exposure with XCI showed similar results as correlation of lysoGb3 to XCI. Findings in the group of late‐onset FD females point to a strong effect of XCI on lysoGb3 exposure by steeply increasing with XCI skewed in favor of expression of mutated allele (Figure 3B).

We used AGAL activity values in WBCs and lysoGb3 in plasma to calculate the ratio [17] and correlate these values with XCI. Integration of these three quantitative biochemical and genetic parameters allowed for a superior categorization of female patients based on their phenotype compared to the individual use of AGAL activity or plasma lysoGb3. The differentiation is suggested both in the plot using continuous scaling of XCI (Figure 4A) and in data categorized by XCI ranges (Figure 4B). A similar pattern is also evident when we normalized the AGAL activity values in WBCs and lysoGb3 in plasma ratio values by XCI to calculate TCM (Figure 4C). Identifiable in the scatterplot shown in Figure 4A, TCM values better demonstrate continuum with partial overlaps rather than strict separation of the three patient groups (Figure 4D).

Linear correlation of datasets in classic and late‐onset FD females also better corresponded to the values in male patients with identical phenotypes. The linear regression plots for the classic and late‐onset FD female groups crossed the XCI axis at 97%–99% of the inactive *wt GLA* allele, which could thus represent a XCI threshold percentage determining the biochemical range seen in male patients. The linear response between the ratio values and XCI was tighter in patients with identical genotypes (e.g., *R* ^2^ = 0.9391 for patients carrying p.(N215S)).

There are several scoring scales to assess FD severity or effects of therapy (MSSI, FOS‐MSSI, DS3, or FASTEX). MSSI is a composite clinical score reflecting cumulative organ involvement and symptom burden. It integrates neurological, renal, cardiovascular, and general clinical domains but does not reflect genetic status, XCI pattern, α‐galactosidase A (AGAL) activity, or lysoGb3 concentration. Disease duration, environmental modifiers, comorbidities, treatment exposure, and age all influence the final score. While lysoGb3 may fluctuate or plateau in time, XCI is (similar to AGAL activity) a relatively stable parameter. Therefore, correlations between XCI and MSSI may become apparent only in specific subgroups depending on age distribution and stage of the disease.

It has been previously reported that overall MSSI scores do not significantly differ between male and female patients [20] and positively correlate with age [5, 16, 25, 26, 34]. Despite links of these scores and XCI and/or biochemical parameters such as AGAL activity or lysoGb3, only a limited number of studies provide quantitative data for all evaluated parameters [25, 26, 30, 32]. Significant differences in MSSI and DS3 between categorized groups of FD females (skewed for *mutant*, random, skewed for *wt*) were documented by Echevaria et al. [26]. Positive correlation between FASTEX score and methylation of normal *GLA* allele was shown by Hossain et al. [32]. A limitation of most of these studies was inclusion of samples from treated patients [26, 30]. To avoid potential bias introduced by therapy, we decided to include only treatment‐naive females. As a result, majority of the total MSSI (and FOS‐MSSI) values were in the mild range of the scale. The age‐related increase of MSSI, FOS‐MSSI and distribution of ΔFOS‐MSSI scores in our cohort were similar to previous studies [21, 22]. Interestingly, ΔFOS‐MSSI values in the females with AGALopathic variants systematically implied better FOS‐MSSI scores than predicted for a given age [22]. Like Echevaria et al. [26] we did not observe significant differences in MSSI values between the three phenotypic patient groups. In contrast to the latter authors, we have, however, identified significantly different MSSI scores only in patients with ≤ 25% of the inactive *wt GLA* allele. Interestingly, the only group with MSSI scores systematically increasing with the inactivation of the *wt GLA* allele were the patients with AGALopathic variants. This observation was also evident in patients with identical genotypes. Contrary to correlations shown for AGAL activity in WBCs, plasma lysoGb3 and their ratio to inactivation of the *wt GLA* allele in Figures 2, 3, 4, MSSI values were not overall correlated with any of these variables.

Clinical progression in patients with AGALopathic *GLA* variants may be more subtly modulated by XCI, leading to an association with MSSI particularly in the lowest ranges of the score. In classic phenotype(s), by contrast, the impacts of profound enzymatic deficiency may dominate the disease expression, potentially masking the incremental effect of XCI. Thus, the absence of correlation in classic and late‐onset groups should be interpreted cautiously and not necessarily as a lack of biological relevance of XCI, but rather as an indicator that additional factors (particularly age‐related cumulative damage) may dilute or overshadow the measurable impact.

There is currently no scoring system that would integrate FD‐specific clinical symptoms with biochemical parameters [21]. Moreover, long‐term follow‐up studies on the natural course of the disease and biomarker levels in untreated FD patients are difficult to conduct due to therapy‐related ethical reasons. Considering these limitations and relatively weak correlation of MSSI to genetic and biochemical markers, we tested the discriminatory efficiency of a single Integrative Clinical‐Laboratory quotient (ICLq) in our cohort of mildly affected females. To do so, we modified MSSI scores to gMSSI+ by adding a value of 1 just for the presence of a *GLA* variant regardless of its impacts. This modification not only avoided zero values of ICLq (gMSSI+/TCM) but also allowed inclusion of asymptomatic (MSSI = 0) individuals. Through MSSI, ICLq reflects the most widely used FD clinical score. AGAL activity, lysoGb3, and XCI, all of which are also incorporated into ICLq, are currently the best available laboratory parameters defining FD in females. As demonstrated in Figure 5, unlike MSSI (or FOS‐MSSI), ICLq divided females in the cohort according to their predicted phenotypes. Furthermore, a calculation of the average annual increase of the quotient in the three groups suggested the potential of ICLq to predict longitudinal change of clinical and laboratory parameters. A parameter like ICLq may, either in this or a modified format (e.g., with differential weighting of *GLA* variants based on their known phenotypic impacts Figure S15), help patient categorization, management, and follow‐up. We also believe that such a complex integrative marker could improve prediction of effects of specific *GLA* variants on clinical penetrance and tested biochemical changes. While we acknowledge that further expert consensus on a marker such as ICLq is needed, it is reasonable to speculate that it could also prove beneficial (in a modified version omitting XCI) also in male FD patients.

Further important, the current concept of FD pathogenesis suggests that certain *GLA* variants may impact not only the enzymatic activity (leading to AGAL deficiency and subsequent lysosomal storage), but may also result in misprocessed AGAL within the early secretory pathway and trigger ER stress and UPR (AGAlopathy). Our study addressed analytical separation of the three phenotypic groups using the currently available biochemical and molecular‐genetic measures; we did not investigate protocols that would effectively quantify the proteinopathic component of FD pathology. Development of robust methodologies for testing AGALopathic impacts and implementation of such tools into clinical‐laboratory diagnostics remain important future challenges.

## 5 Conclusion

To conclude, our study demonstrates that quantitative testing of the three key parameters (AGAL activity in WBCs, lysoGb3 in plasma and XCI) may aid phenotypic categorization of female patients with (pathogenic) *GLA* variants. Although we show positive correlations between AGAL activity and XCI, lysoGb3 and XCI, the strongest discriminative effects were achieved for XCI in relation to the ratio of AGAL activity to lysoGb3 in plasma. Our data also provide additional important cues that individuals carrying *GLA* variants recently suggested as predominantly AGALopathic represent a specific group (in a number of aspects) distinct from patients with classic or late‐onset FD.

## Author Contributions

L.K.: conceptualization, data curation, formal analysis, funding acquisition, investigation, methodology, project administration, supervision, validation, writing – original draft, writing – review and editing. L.D., L.B., M.R., P.S., R.B., and H.P.: conceptualization, data curation, formal analysis, investigation, methodology, project administration, validation, writing – original draft, writing – review and editing. J.L.: conceptualization, investigation, methodology, supervision, validation, writing – original draft, writing – review and editing. P.R.: data curation, writing – original draft, writing – review and editing. G.D.: resources, supervision, writing – original draft, writing – review and editing. S.R.: resources, supervision, writing – original draft, writing – review and editing. A.B.: data curation, writing – original draft, writing – review and editing. A.L.: conceptualization, funding acquisition, project administration, resources, supervision, validation, writing – original draft, writing – review and editing. J.S.: conceptualization, data curation, formal analysis, funding acquisition, supervision, validation, writing – original draft, writing – review and editing.

## Funding

This work was supported by the Ministry of Health of the Czech Republic in cooperation with the Czech Health Research Council under projects nr. NU21‐08–00324 (L.K.) and NW24‐04‐00067 (J.S.) and from the project MULTIOMICS_CZ (Programme Johannes Amos Comenius, Ministry of Education, Youth and Sports of the Czech Republic, ID Project CZ.02.01.01/00/23_020/0008540)—co‐funded by the European Union, by the project National Institute for Research of Metabolic and Cardiovascular Diseases Funded by the European Union Next Generation EU Programme EXCELES, ID Project No. LX22NPO5104 (L.K.), and the institutional programs of Charles University in Prague UNCE/MED/007, Cooperatio/DIAG/1FM and SVV 260631, by MH CZ – DRO VFN64165 and by GZ201711701 grant by Sanofi (A.L.), the study was also supported by Charles University, project GA UK No. 70224.

## Ethics Statement

This study was approved by the ethics committee of General University Hospital in Prague No. 23/20Grant AZV VES 2021 1.LF UK and performed in accordance with the Helsinki declaration. This is part of the study conditions approved by the ethics committee.

## Consent

We confirm that all material collection and analyses were done after written informed consent from patients was obtained.

## Conflicts of Interest

A.L. received consulting and speaker's honoraria from Sanofi, Amicus Therapeutucs and Chiesi. G.D. received consulting and speaker's honoraria from Takeda, Sanofi, Amicus Therapeutucs and Chiesi. S.R. received consulting and speaker's honoraria from Takeda, Sanofi and Amicus Therapeutucs. A.L. received the institutional support by GZ201711701 grant by Sanofi.

## Supporting information


**Table S1:** MSSI and MSSI‐derived scores—total and in individual categories.
**Figure S1:** Age and correlation of age and XCI in the patient cohort.
**Figure S2:** Correlation of AGAL activity in plasma to XCI.
**Figure S3:** Correlation of lysoGb3 exposure with XCI in late‐onset FD females.
**Figure S4:** Correlation of biochemical parameters to XCI in patients with selected GLA variants.
**Figure S5:** MSSI, observed FOS‐MSSI, and ΔFOS‐MSSI scores in the three patient groups.
**Figure S6:** Correlation of MSSI, (observed) FOS‐MSSI, and ΔFOS‐MSSI scores with age of the patients.
**Figure S7:** Correlation of MSSI and ΔFOS‐MSSI scores with XCI in the patients.
**Figure S8:** Correlation of MSSI and ΔFOS‐MSSI to XCI in selected GLA variants.
**Figure S9:** Correlation of MSSI values with AGAL activity in WBCs.
**Figure S10:** Correlation of MSSI values with lysoGb3 in plasma.
**Figure S11:** Correlation of MSSI values with AGAL in WBCs/lysoGb3 in plasma.
**Figure S12:** Correlation of biochemical parameters to MSSI in selected GLA variants.
**Figure S13:** Correlation of biochemical parameters to ∆FOS‐MSSI in selected GLA variants.
**Figure S14:** Correlation of lysoGb3 in plasma to age in selected GLA variants.
**Figure S15:** Integrative clinical‐laboratory FD quotient in females with gMSSI+ scores using weighted GLA variant values.

## Data Availability

The data that support the findings of this study are available from the corresponding author upon reasonable request.
