# Supplementary material for: Tri‐Parametric Assessment of α‐Galactosidase A Activity, lysoGb3 and X‐Inactivation Aids Genotype‐Phenotype Categorization of Fabry Disease Female Patients
Source: J Inherit Metab Dis. 2026 Jun 22;49(4):e70218. doi: 10.1002/jimd.70218 (PMC13284639; doi:10.1002/jimd.70218)
Supplement: Supplementary file 1 — Table S1: MSSI and MSSI‐derived scores—total and in individual categories. Figure S1: Age and correlation of age and XCI in the patient cohort. Figure S2: Correlation of AGAL activity in plasma to XCI. Figure S3: Correlation of lysoGb3 exposure with XCI in late‐onset FD females. Figure S4: Correlation of biochemical parameters to XCI in patients with selected GLA variants. Figure S5: MSSI, observed FOS‐MSSI, and ΔFOS‐MSSI scores in the three patient groups. Figure S6: Correlation of MSSI, (observed) FOS‐MSSI, and ΔFOS‐MSSI scores with age of the patients. Figure S7: Correlation of MSSI and ΔFOS‐MSSI scores with XCI in the patients. Figure S8: Correlation of MSSI and ΔFOS‐MSSI to XCI in selected GLA variants. Figure S9: Correlation of MSSI values with AGAL activity in WBCs. Figure S10: Correlation of MSSI values with lysoGb3 in plasma. Figure S11: Correlation of MSSI values with AGAL in WBCs/lysoGb3 in plasma. Figure S12: Correlation of biochemical parameters to MSSI in selected GLA variants. Figure S13: Correlation of biochemical parameters to ∆FOS‐MSSI in selected GLA variants. Figure S14: Correlation of lysoGb3 in plasma to age in selected GLA variants. Figure S15: Integrative clinical‐laboratory FD quotient in females with gMSSI+ scores using weighted GLA variant values. [file JIMD-49-0-s001.docx]

**Supplementary material**

**LysoGb3 (life) exposure calculation**

Example of the calculation is provided for the group of patients with classic FD. Groups of patients with late-onset FD and patients carrying AGALopathic *GLA* variants were processed identically.

Female patients were divided into two groups – those with lysoGb3 above the intercept value (blue dots) of the regression line equation (black dotted line) and those with lysoGb3 equal or below the intercept value (green dots).

Calculation for the green dots was based on the following equation:

$$life exposure=lysoGb3\cdot age$$


Life exposure (for one selected patient – green dot) is represented by the gray region.

Calculation for the blue dots was based on the following equation:

$$life exposure=\left( intercept\cdot age \right)+\frac{\left( lysoGb3-intercept \right)\cdot age}{2}$$


Life exposure (for one selected patient - blue dot) is represented by the gray region.

***Table S1 – MSSI and MSSI-derived scores – total and in individual categories***

| **No.** | **Age (years)** | **MSSI Total** | **Observed FOS-MSSI total** | **Predicted FOS-MSSI total** | **∆ FOS-MSSI** | **Face** | **Angiokeratoma** | **Oedema** | **Muculoskeletal** | **Cornea verticillata** | **Diaphoresis** | **Abdominal pain** | **Diarrhea/constipation** | **Hemorrhoids** | **Pulmonary** | **NYHA class** | **Tinnitus** | **Vertigo** | **Acroparesthesia** | **Fever pain crisis** | **Cerebrovascular** | **Depression** | **Fatigue** | **Reduced activity** | **LVH** | **Valves** | **ECG** | **Pacemaker** | **Hypertension** | **Renal dysfunction** |
| --- | --- | --- | --- | --- | --- | --- | --- | --- | --- | --- | --- | --- | --- | --- | --- | --- | --- | --- | --- | --- | --- | --- | --- | --- | --- | --- | --- | --- | --- | --- |
| 1 | 61 | 3 | 4,5 | 16,1 | -11,6 | 0 | 1(1,5) | 0 | 0 | 0 | 0 | 0 | 0 | 0 | 0 | 1(0) | 0 | 0 | 0 | 0 | 1(3) | 0 | 0 | 0 | 0 | 0 | 0 | 0 | 0 | 0 |
| 2 | 38 | 3 | 3,0 | 8,2 | -5,2 | 0 | 0 | 0 | 0 | 1 | 0 | 0 | 0 | 0 | 0 | 0 | 0 | 0 | 0 | 0 | 0 | 0 | 0 | 0 | 0 | 0 | 2 | 0 | 0 | 0 |
| 3 | 34 | 11 | 11,5 | 7,1 | 4,4 | 0 | 1(1,5) | 1 | 0 | 1 | 1 | 0 | 0 | 0 | 2 | 1(0) | 0 | 1 | 3(4) | 0 | 0 | 0 | 0 | 0 | 0 | 0 | 0 | 0 | 0 | 0 |
| 4 | 38 | 1 | 0,0 | 8,2 | -8,2 | 0 | 0 | 0 | 0 | 0 | 0 | 0 | 0 | 0 | 0 | 1(0) | 0 | 0 | 0 | 0 | 0 | 0 | 0 | 0 | 0 | 0 | 0 | 0 | 0 | 0 |
| 5 | 46 | 18 | 18,5 | 10,6 | 7,9 | 0 | 2(1,5) | 1 | 1 | 1 | 1 | 2 | 1 | 0 | 0 | 0 | 1 | 0 | 3(4) | 0 | 0 | 0 | 0 | 0 | 0 | 0 | 0 | 0 | 1 | 4 |
| 6 | 35 | 7 | 7,0 | 7,3 | -0,3 | 0 | 0 | 0 | 0 | 1 | 0 | 0 | 0 | 0 | 0 | 0 | 0 | 0 | 0 | 0 | 0 | 0 | 0 | 0 | 0 | 0 | 2 | 0 | 0 | 4 |
| 7 | 49 | 21 | 24,0 | 11,6 | 12,4 | 0 | 0 | 0 | 1 | 1 | 1 | 0 | 0 | 0 | 0 | 0 | 1 | 1 | 3(4) | 2(0) | 1(3) | 0 | 1(0) | 1(0) | 8(10) | 0 | 2 | 0 | 0 | 0 |
| 8 | 19 | 5 | 5,0 | 3,6 | 1,4 | 0 | 0 | 0 | 0 | 0 | 0 | 0 | 1 | 0 | 0 | 1(0) | 0 | 0 | 3(4) | 0 | 0 | 0 | 0 | 0 | 0 | 0 | 0 | 0 | 0 | 0 |
| 9 | 56 | 6 | 5,0 | 14,1 | -9,1 | 0 | 0 | 0 | 0 | 1 | 0 | 2 | 1 | 0 | 0 | 0 | 0 | 0 | 0 | 0 | 0 | 0 | 0 | 0 | 1(0) | 0 | 0 | 0 | 1 | 0 |
| 10 | 37 | 19 | 17,0 | 7,9 | 9,1 | 0 | 0 | 0 | 0 | 0 | 0 | 0 | 0 | 0 | 0 | 0 | 0 | 0 | 0 | 0 | 0 | 0 | 0 | 0 | 12(10) | 0 | 2 | 0 | 1 | 4 |
| 11 | 63 | 19 | 22,5 | 16,9 | 5,6 | 0 | 2(1,5) | 0 | 0 | 0 | 0 | 0 | 0 | 0 | 0 | 2 | 0 | 0 | 0 | 0 | 1(3) | 0 | 0 | 0 | 8(10) | 0 | 2 | 0 | 0 | 4 |
| 12 | 29 | 7 | 10,0 | 5,8 | 4,2 | 0 | 0 | 0 | 0 | 1 | 0 | 0 | 0 | 0 | 0 | 0 | 0 | 0 | 0 | 0 | 3(6) | 0 | 0 | 0 | 0 | 0 | 2 | 0 | 1 | 0 |
| 13 | 22 | 1 | 1,0 | 4,2 | -3,2 | 0 | 0 | 0 | 0 | 1 | 0 | 0 | 0 | 0 | 0 | 0 | 0 | 0 | 0 | 0 | 0 | 0 | 0 | 0 | 0 | 0 | 0 | 0 | 0 | 0 |
| 14 | 23 | 9 | 8,0 | 4,5 | 3,5 | 0 | 0 | 0 | 0 | 0 | 0 | 2 | 1 | 0 | 2 | 2 | 0 | 0 | 0 | 0 | 0 | 1 | 0 | 1(0) | 0 | 0 | 0 | 0 | 0 | 0 |
| 15 | 18 | 17 | 17,0 | 3,5 | 13,5 | 0 | 0 | 0 | 0 | 0 | 0 | 2 | 0 | 0 | 2 | 2 | 1 | 2(1) | 3(4) | 0 | 0 | 1 | 1(0) | 1(0) | 0 | 0 | 0 | 0 | 0 | 4 |
| 16 | 31 | 15 | 16,0 | 6,3 | 9,7 | 0 | 0 | 0 | 1 | 0 | 0 | 2 | 0 | 0 | 0 | 3(2) | 0 | 1 | 3(4) | 0 | 5(6) | 0 | 1(0) | 1(0) | 0 | 0 | 0 | 0 | 0 | 0 |
| 17 | 31 | 0 | 0,0 | 6,3 | -6,3 | 0 | 0 | 0 | 0 | 0 | 0 | 0 | 0 | 0 | 0 | 0 | 0 | 0 | 0 | 0 | 0 | 0 | 0 | 0 | 0 | 0 | 0 | 0 | 0 | 0 |
| 18 | 75 | 20 | 18,0 | 22,2 | -4,2 | 0 | 0 | 0 | 0 | 0 | 0 | 0 | 0 | 0 | 0 | 2 | 0 | 0 | 0 | 0 | 1(3) | 0 | 1(0) | 1(0) | 12(10) | 1 | 2 | 0 | 0 | 0 |
| 19 | 23 | 5 | 6,0 | 4,5 | 1,5 | 0 | 0 | 0 | 0 | 0 | 0 | 0 | 0 | 0 | 0 | 0 | 0 | 0 | 3(4) | 0 | 0 | 0 | 0 | 0 | 0 | 0 | 2 | 0 | 0 | 0 |
| 20 | 33 | 0 | 0,0 | 6,8 | -6,8 | 0 | 0 | 0 | 0 | 0 | 0 | 0 | 0 | 0 | 0 | 0 | 0 | 0 | 0 | 0 | 0 | 0 | 0 | 0 | 0 | 0 | 0 | 0 | 0 | 0 |
| 21 | 35 | 2 | 0,0 | 7,3 | -7,3 | 0 | 0 | 0 | 0 | 0 | 0 | 0 | 0 | 0 | 0 | 0 | 0 | 0 | 0 | 0 | 0 | 0 | 1(0) | 1(0) | 0 | 0 | 0 | 0 | 0 | 0 |
| 22 | 40 | 4 | 6,0 | 8,8 | -2,8 | 0 | 0 | 0 | 0 | 0 | 0 | 0 | 0 | 0 | 0 | 1(0) | 0 | 0 | 0 | 0 | 3(6) | 0 | 0 | 0 | 0 | 0 | 0 | 0 | 0 | 0 |
| 23 | 20 | 17 | 17,0 | 3,8 | 13,2 | 0 | 0 | 0 | 0 | 1 | 0 | 0 | 0 | 0 | 0 | 0 | 0 | 0 | 6 | 0 | 1(3) | 1 | 1(0) | 1(0) | 0 | 0 | 2 | 0 | 0 | 4 |
| 24 | 68 | 17 | 19,0 | 19,0 | 0,0 | 0 | 0 | 0 | 0 | 0 | 0 | 0 | 0 | 0 | 0 | 2 | 0 | 0 | 0 | 0 | 0 | 0 | 0 | 0 | 8(10) | 0 | 2 | 0 | 1 | 4 |
| 25 | 47 | 2 | 2,0 | 11,0 | -9,0 | 0 | 0 | 0 | 0 | 0 | 0 | 0 | 0 | 0 | 0 | 2 | 0 | 0 | 0 | 0 | 0 | 0 | 0 | 0 | 0 | 0 | 0 | 0 | 0 | 0 |
| 26 | 36 | 0 | 0,0 | 7,6 | -7,6 | 0 | 0 | 0 | 0 | 0 | 0 | 0 | 0 | 0 | 0 | 0 | 0 | 0 | 0 | 0 | 0 | 0 | 0 | 0 | 0 | 0 | 0 | 0 | 0 | 0 |
| 27 | 71 | 19 | 19,0 | 20,3 | -1,3 | 0 | 0 | 0 | 0 | 0 | 0 | 0 | 0 | 0 | 0 | 2 | 0 | 0 | 0 | 0 | 0 | 0 | 1(0) | 1(0) | 8(10) | 0 | 2 | 0 | 1 | 4 |
| 28 | 64 | 19 | 17,0 | 17,3 | -0,3 | 0 | 0 | 0 | 0 | 0 | 0 | 0 | 0 | 0 | 0 | 0 | 0 | 0 | 0 | 0 | 0 | 0 | 0 | 0 | 12(10) | 0 | 2 | 0 | 1 | 4 |
| 29 | 28 | 5 | 5,0 | 5,6 | -0,6 | 0 | 0 | 0 | 0 | 0 | 1 | 0 | 0 | 0 | 0 | 0 | 0 | 0 | 0 | 0 | 0 | 0 | 0 | 0 | 0 | 0 | 0 | 0 | 0 | 4 |
| 30 | 58 | 23 | 22,0 | 14,9 | 7,1 | 0 | 0 | 0 | 1 | 0 | 0 | 0 | 0 | 0 | 0 | 2 | 0 | 0 | 3(4) | 0 | 0 | 0 | 1(0) | 1(0) | 0 | 0 | 2 | 4 | 1 | 8 |
| 31 | 19 | 2 | 2,5 | 3,6 | -1,1 | 0 | 1(1,5) | 0 | 0 | 0 | 1 | 0 | 0 | 0 | 0 | 0 | 0 | 0 | 0 | 0 | 0 | 0 | 0 | 0 | 0 | 0 | 0 | 0 | 0 | 0 |
| 32 | 42 | 4 | 4,0 | 9,4 | -5,4 | 0 | 0 | 0 | 0 | 0 | 0 | 0 | 0 | 0 | 0 | 0 | 0 | 0 | 0 | 0 | 0 | 0 | 0 | 0 | 0 | 0 | 0 | 0 | 0 | 4 |
| 33 | 38 | 2 | 2,0 | 8,2 | -6,2 | 0 | 0 | 0 | 0 | 0 | 0 | 0 | 0 | 0 | 0 | 0 | 0 | 0 | 0 | 0 | 0 | 0 | 0 | 0 | 0 | 0 | 1 | 0 | 1 | 0 |
| 34 | 46 | 0 | 0,0 | 10,6 | -10,6 | 0 | 0 | 0 | 0 | 0 | 0 | 0 | 0 | 0 | 0 | 0 | 0 | 0 | 0 | 0 | 0 | 0 | 0 | 0 | 0 | 0 | 0 | 0 | 0 | 0 |
| 35 | 68 | 6 | 7,0 | 19,0 | -12,0 | 0 | 0 | 0 | 0 | 0 | 0 | 0 | 0 | 0 | 0 | 2 | 0 | 0 | 0 | 0 | 1(3) | 1 | 0 | 0 | 1(0) | 0 | 0 | 0 | 1 | 0 |
| 36 | 30 | 6 | 6,0 | 6,1 | -0,1 | 0 | 0 | 0 | 0 | 0 | 0 | 0 | 0 | 0 | 0 | 2 | 0 | 0 | 0 | 0 | 0 | 0 | 0 | 0 | 0 | 0 | 0 | 0 | 0 | 4 |
| 37 | 21 | 3 | 3,0 | 4,0 | -1,0 | 0 | 0 | 0 | 0 | 0 | 0 | 2 | 1 | 0 | 0 | 0 | 0 | 0 | 0 | 0 | 0 | 0 | 0 | 0 | 0 | 0 | 0 | 0 | 0 | 0 |
| 38 | 35 | 1 | 3,0 | 7,3 | -4,3 | 0 | 0 | 0 | 0 | 0 | 0 | 0 | 0 | 0 | 0 | 0 | 0 | 0 | 0 | 0 | 1(3) | 0 | 0 | 0 | 0 | 0 | 0 | 0 | 0 | 0 |
| 39 | 30 | 3 | 6,0 | 6,1 | -0,1 | 0 | 0 | 0 | 0 | 0 | 0 | 0 | 0 | 0 | 0 | 0 | 0 | 0 | 0 | 0 | 3(6) | 0 | 0 | 0 | 0 | 0 | 0 | 0 | 0 | 0 |
| 40 | 39 | 18 | 17,0 | 8,5 | 8,5 | 0 | 0 | 0 | 0 | 0 | 0 | 2 | 1 | 0 | 2 | 0 | 0 | 2(1) | 3(4) | 2(0) | 5(6) | 1 | 0 | 0 | 0 | 0 | 0 | 0 | 0 | 0 |
| 41 | 46 | 0 | 0,0 | 10,6 | -10,6 | 0 | 0 | 0 | 0 | 0 | 0 | 0 | 0 | 0 | 0 | 0 | 0 | 0 | 0 | 0 | 0 | 0 | 0 | 0 | 0 | 0 | 0 | 0 | 0 | 0 |
| 42 | 56 | 0 | 0,0 | 14,1 | -14,1 | 0 | 0 | 0 | 0 | 0 | 0 | 0 | 0 | 0 | 0 | 0 | 0 | 0 | 0 | 0 | 0 | 0 | 0 | 0 | 0 | 0 | 0 | 0 | 0 | 0 |
| 43 | 28 | 7 | 7,0 | 5,6 | 1,4 | 0 | 0 | 0 | 0 | 0 | 0 | 0 | 0 | 0 | 0 | 0 | 0 | 0 | 0 | 0 | 0 | 1 | 0 | 0 | 0 | 0 | 2 | 0 | 0 | 4 |
| 44 | 56 | 8 | 6,0 | 14,1 | -8,1 | 0 | 0 | 0 | 0 | 0 | 0 | 2 | 1 | 0 | 0 | 0 | 1 | 1 | 0 | 0 | 0 | 1 | 1(0) | 1(0) | 0 | 0 | 0 | 0 | 0 | 0 |
| 45 | 51 | 1 | 1,0 | 12,3 | -11,3 | 0 | 0 | 0 | 0 | 0 | 0 | 0 | 0 | 0 | 0 | 0 | 0 | 0 | 0 | 0 | 0 | 0 | 0 | 0 | 0 | 0 | 1 | 0 | 0 | 0 |
| 46 | 51 | 0 | 0,0 | 12,3 | -12,3 | 0 | 0 | 0 | 0 | 0 | 0 | 0 | 0 | 0 | 0 | 0 | 0 | 0 | 0 | 0 | 0 | 0 | 0 | 0 | 0 | 0 | 0 | 0 | 0 | 0 |
| 47 | 39 | 18 | 18,0 | 8,5 | 9,5 | 0 | 0 | 0 | 0 | 0 | 1 | 0 | 0 | 0 | 0 | 2 | 0 | 1 | 0 | 0 | 0 | 1 | 1(0) | 1(0) | 8(10) | 1 | 2 | 0 | 0 | 0 |
| 48 | 29 | 0 | 0,0 | 5,8 | -5,8 | 0 | 0 | 0 | 0 | 0 | 0 | 0 | 0 | 0 |  | 0 | 0 | 0 | 0 | 0 | 0 | 0 | 0 | 0 | 0 | 0 | 0 | 0 | 0 | 0 |
| 49 | 50 | 6 | 7,0 | 12,0 | -5,0 | 0 | 0 | 0 | 0 | 0 | 0 | 0 | 0 | 0 | 0 | 0 | 0 | 0 | 0 | 0 | 5(6) | 1 | 0 | 0 | 0 | 0 | 0 | 0 | 0 | 0 |
| 50 | 49 | 15 | 14,5 | 11,6 | 2,9 | 0 | 1(1,5) | 0 | 0 | 0 | 0 | 2 | 0 | 0 | 0 | 0 | 0 | 0 | 0 | 0 | 5(6) | 1 | 1(0) | 1(0) | 0 | 0 | 0 | 0 | 0 | 4 |
| 51 | 60 | 16 | 17,5 | 15,7 | 1,8 | 1(0) | 1(1,5) | 0 | 0 | 1 | 2 | 0 | 0 | 0 | 0 | 0 | 1 | 1 | 3(4) | 0 | 5(6) | 0 | 0 | 0 | 0 | 0 | 0 | 0 | 1 | 0 |

*Patient numbers and ages correspond to Table 1. MSSI scores were assessed in indvidual patients according to Whybra et al.^1^. “Observed FOS-MSSI” scores were calculated according to Whybra et al.^2^. “Predicted FOS-MSSI” total scores were calculated according to Hughes et al.^3^. ΔFOS-MSSI = (Observed FOS-MSSI) – (Predicted FOS-MSSI) in individual patients. Positive ΔFOS-MSSI values imply higher FOS-MSSI scores than predicted by Hughes et al.^3^ Negative ΔFOS-MSSI values imply lower FOS-MSSI scores than predicted by Hughes et al.^3^*

*MSSI scores are shown in individual categories. If different from MSSI scores, “Observed FOS-MSSI” scores are listed in brackets.*

**A**

**B**

***Figure S1 - Age and correlation of age and XCI in the patient cohort***

*(****A****) Distribution of ages in individual patient groups. No statistically significant differences were observed.*

*(****B****) WBC XCI values correlated to age of individual patients in the cohort. Blue box represents the range of random XCI. Overall, 21 (41.2%) females presented with skewed XCI. Patient numbers correspond to Table 1. XCI and age do not systematically (overall (black dotted line) or in individual groups) correlate. Note, however, the cluster of late-onset FD females (#18, #24, #27, #28 and #35) aged >65 years with skewed XCI.*

***Figure S2 - Correlation of AGAL activity in plasma to XCI***

*AGAL values in plasma are substantially less correlated to XCI than AGAL activity values in WBCs (R^2^=0.5429, Figure 2). Patient numbers correspond to Table 1 and Table S1. Vertical blue lines highlight 25% and 75% of inactive wt GLA allele.*

***Figure S3 - Correlation of lysoGb3 exposure with XCI in late-onset FD females***

*Lifetime lysoGb3 exposure values steeply increase in the range of XCI skewed toward the active mutated GLA allele.*

*Contribution of age to the calculated values can be demonstrated in patients #24-27. These four patients share the same GLA variant, however, patients #25 and #26 (below the green line) were ~28 years younger than patients #24 and #27 (above the green line).*

*Vertical blue line highlights 75 % of the inactive wt GLA allele. The horizontal dashed green line represents the maximum exposure calculated among patients with AGALopathic GLA variants.*

***Figure S4 – Correlation of biochemical parameters to XCI in patients with selected GLA variants***

*Correlation of AGAL activity in WBCs (****A****,* ***D****,* ***G****,* ***J****, and* ***M****), lysoGb3 in plasma (****B****,* ***E****,* ***H****,* ***K****, and* ***N****), and AGAL_WBCs/lysoGb3_plasma ratio (****C****,* ***F****,* ***I****,* ***L****, and* ***O****) with XCI in late-onset FD patients (****A****-****F****) with GLA variants c.644A>G p.(N215S) and c.801+48T>G p.(L268Vfs*4) and in patients with AGALopathic GLA variants (****G****-****O****) c.1181T>G p.(L394P) and c.427G>A p.(A143T), and c.937G>T p.(D313Y).*

*Relatively tight correlation of AGAL activity in WBCs/lysoGb3 in plasma ratio to XCI was detected for all five GLA variants. Summary data for all patients and GLA variants in the studied cohort are provided in Figures 2-4.*

***Figure S5 – MSSI, Observed FOS-MSSI, and ΔFOS-MSSI scores in the three patient groups***

*(****A****) Tight correlation (R^2^=0.9706) of MSSI and Observed FOS-MSSI scores in individual patients.*

*(****B, C****) There are no statistically significant differences in MSSI and Observed FOS-MSSI scores between the three tested patient groups by the unpaired two-tailed Student t-test. Average (× ) and median (horizontal lines) values are ordered classic>late-onset>AGALopathic patient groups.*

*(****D****) There is no statistically significant difference in ΔFOS-MSSI scores (Observed FOS-MSSI – Predicted FOS-MSSI) between the three tested patient groups by the unpaired two-tailed Student t-test. Average (× ) and median (horizontal lines) values are positive in the group of FD patients with classic GLA variants, close to zero in patients with late-onset GLA variants, and negative in patients with AGALopathic GLA variants.*

***Figure S6 - Correlation of MSSI, (observed) FOS-MSSI, and ΔFOS-MSSI scores with age of the patients***

*Color coding of the three phenotypic groups and positions of the individual patients in the scatter-plot are maintained in all panels. Black dotted line corresponds to the overall (across all three phenotypic groups) regression.*

*(****A****) MSSI values tend to increase with age in all three patient groups*

*(****B****) XCI (% of inactive GLA allele) values divided into the three ranges are graphically coded for each patient. Full (≥75%) and dotted (≤25%) lines plot regression in patients with skewed XCI. Dashed line reflects regression calculated in patients with random XCI. All females with XCI of the wt allele ≤25% (empty circles*) *have, regardless of age, low MSSI values. The only exception is female #30 with late-onset phenotype. Compare to Figure S7.*

*The increase of MSSI with age seen in patients with random XCI of all three phenotypic groups corresponds to results reported earlier by other authors^1,4,5,6,7^. XCI values in individual patients are summarized in Table 1.*

*(****C****) Observed FOS-MSSI scores increase similarly to MSSI and follow distribution reported earlier by Hughes et al^3^*

*(****D****) ΔFOS-MSSI* scores were distributed similar to previous study by Hughes et al.^3^ Lower observed MSSI scores in comparison to predicted MSSI scores (negative ΔFOS-MSSI scores) were detected particularly in older patients with AGALopathic variants.

***Figure S7 - Correlation of MSSI and ΔFOS-MSSI scores with XCI in the patients***

*(****A****)* *regression plots are shown for individual patient groups. The overall regression is depicted by the dotted line. Vertical blue lines highlight 25 and 75 % of inactive wt GLA allele. A tighter correlation was detected only in patients carrying AGALopathic GLA variants.*

*(****B****)* *Unlike to Ecchevaria et al.^3^, MSSI values were statistically different between the patients with ≤25% of inactive wt allele and patients with random XCI and ≥75% of inactive wt allele. The two latter groups do not differ from each other.* ******* *- unpaired one-tailed Student t-test p<0.05*

*(****C****) Similar to data shown in (****A****)* ΔFOS-MSSI scores tend to increase (R^2^=0.526) with XCI only in the AGALopathic group. Important, females with XCI values favoring active wt allele have negative ΔFOS-MSSI scores.

*For further details see Figure S8.*

***Figure S8 – Correlation of MSSI and ΔFOS-MSSI to XCI in selected GLA variants***

*Detailed correlation plots of MSSI and* ***ΔFOS-MSSI*** *with XCI in late-onset FD patients (****A,*** ***B, F, G****) with GLA variants c.644A>G p.(N215S) and c.801+48T>G p.(L268Vfs*4) and in patients with AGALopathic GLA variants (****C****-****E, H-J****) c.1181T>G p.(L394P) and c.427G>A p.(A143T), and c.937G>T p.(D313Y). The tightest correlation was detected for AGALopathic variants c.1181T>G p.(L394P) and c.427G>A p.(A143T).*

***Figure S9 - Correlation of MSSI values with AGAL activity in WBCs***

*Color coding of the three phenotypic groups and positions of the individual patients in the scatter-plot are maintained in panels* ***A*** *and* ***B****. (****A****) Black dotted line corresponds to the overall (across all three groups) regression. (****B****) XCI values divided into the three ranges are shown for each patient. Full (≥75%) and dotted (≤25%) lines plot regression in patients with skewed XCI. Dashed line reflects regression calculated in patients with random XCI.*

*The decrease of MSSI with increased AGAL activity in WBCs seen in patients with AGALopathic GLA variants (****A****) is paralleled by XCI values in the range ≤25% of inactivated wt allele in these patients (****B****).*

*For further details see Figure S12.*

***Figure S10 - Correlation of MSSI values with lysoGb3 in plasma***

*Color coding of the three phenotypic groups and positions of the individual patients in the scatter-plot are maintained in panels* ***A*** *and* ***B****. (****A****) Black dotted line corresponds to the overall (across all three groups) regression. (****B****) XCI values divided into the three ranges are graphically coded for each patient. Full (≥75%) and dotted (≤25%) lines plot regression in patients with skewed XCI. Dashed line reflects regression calculated in patients with random XCI.*

*For further details see Figure S12.*

***Figure S11 - Correlation of MSSI values with AGAL in WBCs/lysoGb3 in plasma***

*Color coding of the three phenotypic groups and positions of the individual patients in the scatter-plot are maintained in panels* ***A*** *and* ***B****. (****A****) Black dotted line in* ***A*** *corresponds to the overall (across all three groups) regression. (****B****) XCI values divided into the three ranges are shown for each patient. Full (≥75%) and dotted (≤25%) lines plot regression in patients with skewed XCI. Dashed line reflects regression calculated in patients with random XCI.*

*For further details see Figure S12.*

***Figure S12 – Correlation of biochemical parameters to MSSI in selected GLA variants***

*Detailed correlation of AGAL activity in WBCs (****A****,* ***D****,* ***G****,* ***J****, and* ***M****), lysoGb3 in plasma (****B****,* ***E****,* ***H****,* ***K****, and* ***N****), and AGAL in WBCs/lysoGb3 in plasma ratio (****C****,* ***F****,* ***I****,* ***L****, and* ***O****) and MSSI in late-onset FD patients (****A****-****F****) with GLA variants c.644A>G p.(N215S) and c.801+48T>G p.(L268Vfs*4) and in patients with AGALopathic GLA variants (****G****-****O****) c.1181T>G p.(L394P) and c.427G>A p.(A143T), and c.937G>T p.(D313Y).*

*MSSI tends to decrease with the ratio values in patients with AGALopathic variants.*

***Figure S13 – Correlation of biochemical parameters to ∆FOS-MSSI in selected GLA variants***

*Detailed correlation of AGAL activity in WBCs (****A****,* ***D****,* ***G****,* ***J****, and* ***M****), lysoGb3 in plasma (****B****,* ***E****,* ***H****,* ***K****, and* ***N****), and AGAL in WBCs/lysoGb3 in plasma ratio (****C****,* ***F****,* ***I****,* ***L****, and* ***O****) in late-onset FD patients (****A****-****F****) with GLA variants c.644A>G p.(N215S) and c.801+48T>G p.(L268Vfs*4) and in patients with AGALopathic GLA variants (****G****-****O****) c.1181T>G p.(L394P) and c.427G>A p.(A143T), and c.937G>T p.(D313Y).*

*Similar to MSSI scores, ∆FOS-MSSI scores also tend to decrease with ratio values in patients with AGALopathic variants.*

***Figure S14 – Correlation of lysoGb3 in plasma to age in selected GLA variants***

*Detailed correlation plots of lysoGb3 in plasma and AGAL in WBCs/lysoGb3 in plasma with age in late-onset FD patients (****A,*** ***B****) with GLA variants c.644A>G p.(N215S) and c.801+48T>G p.(L268Vfs*4) and in patients with AGALopathic GLA variants (****C****-****E****) c.1181T>G p.(L394P) and c.427G>A p.(A143T), and c.937G>T p.(D313Y).*

***Figure S15 – Integrative clinical-laboratory FD quotient in females with gMSSI+ scores using weighted GLA variant values.***

*Contrary to Figure 5 which shows ICLq values using gMSSI+ scores attained by adding a value of 1 for the presence of a GLA variant to all original MSSI scores, ICLq values in this Supplementary Figure use gMSSI+ scores gained by increasing the original MSSI values by 1 point for AGALopathic variant, 2 points for late-onset FD associated GLA variants and 3 points for classic FD associated GLA variants. Integrated Clinical Laboratory Quotient (ICLq) was calculated as gMSSI+/TCM (****A****). Average annual increase of ICLq for a given age (ICLq/age) (****B****).*

******* *- unpaired two tailed Student t-test p<0.05,* ******** *- unpaired two tailed Student t-test p<0.01, ********** *- unpaired two tailed Student t-test p<0.001*

**References**

1. Whybra C, Kampmann C, Krummenauer F, et al, "The Mainz Severity Score Index: a new instrument for quantifying the Anderson-Fabry disease phenotype, and the response of patients to enzyme replacement therapy," *Clin Genet* 65, no. 4 (2004): 299-307.

2. Whybra C, Bahner F, Baron K Measurement of disease severity and progression in Fabry disease. In Mehta A, Beck M, Sunder-Plassmann G eds. *Fabry Disease: Perspectives from 5 Years of FOS* Oxford;2006.

3. Hughes DA, Ramaswami U, Barba Romero MA, Deegan P, Investigators FOS, "Age adjusting severity scores for Anderson-Fabry disease," *Mol Genet Metab* 101, no. 2-3 (2010): 219-227.

4. Dobrovolny R, Dvorakova L, Ledvinova J, et al, "Relationship between X-inactivation and clinical involvement in Fabry heterozygotes. Eleven novel mutations in the alpha-galactosidase A gene in the Czech and Slovak population," *J Mol Med (Berl)* 83, no. 8 (2005): 647-654.

5. Echevarria L, Benistan K, Toussaint A, et al, "X-chromosome inactivation in female patients with Fabry disease," *Clin Genet* 89, no. 1 (2016): 44-54.

6. Hossain MA, Wu C, Yanagisawa H, Miyajima T, Akiyama K, Eto Y, "Future clinical and biochemical predictions of Fabry disease in females by methylation studies of the GLA gene," *Mol Genet Metab Rep* 20, no. (2019): 100497.

7. Parini R, Rigoldi M, Santus F, et al, "Enzyme replacement therapy with agalsidase alfa in a cohort of Italian patients with Anderson-Fabry disease: testing the effects with the Mainz Severity Score Index," *Clin Genet* 74, no. 3 (2008): 260-266.
